# 2-(4-Bromo­phen­yl)-2-oxoethyl 4-hy­droxy­benzoate

**DOI:** 10.1107/S1600536811040311

**Published:** 2011-10-05

**Authors:** Hoong-Kun Fun, Wan-Sin Loh, B. Garudachari, Arun M. Isloor, M. N. Satyanarayana

**Affiliations:** a X-ray Crystallography Unit, School of Physics, Universiti Sains Malaysia, 11800 USM, Penang, Malaysia; b Organic Electronics Division, Department of Chemistry, National Institute of Technology–Karnataka, Surathkal, Mangalore 575 025, India; c Department of Physics, National Institute of Technology–Karnataka, Surathkal, Mangalore 575 025, India

## Abstract

In the title compound, C_15_H_11_BrO_4_, the dihedral angle between the aromatic rings is 66.77 (8)°. In the crystal, O—H⋯O, C—H⋯Br and C—H⋯O hydrogen bonds link the mol­ecules, forming layers lying parallel to (101). The crystal packing is further consolidated by C—H⋯π inter­actions and π–π stacking inter­actions [centroid–centroid distance = 3.5476 (7) Å].

## Related literature

For a related structure and background references to phenacyl benzoates, see: Fun *et al.* (2011 ▶). For the synthesis, see: Lund & Langvad (1932 ▶). For a related structure, see: Jin *et al.* (2008 ▶). For the stability of the temperature controller used for the data collection, see: Cosier & Glazer (1986 ▶).
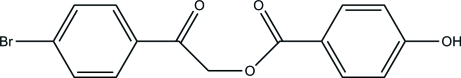


## Experimental

### 

#### Crystal data

C_15_H_11_BrO_4_
*M* *_r_* = 335.15
Monoclinic, 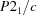

*a* = 6.2917 (2) Å
*b* = 7.7893 (2) Å
*c* = 26.7497 (8) Å
β = 98.234 (2)°
*V* = 1297.43 (7) Å^3^
*Z* = 4
Mo *K*α radiation
μ = 3.18 mm^−1^
*T* = 100 K
0.56 × 0.27 × 0.23 mm

#### Data collection

Bruker SMART APEXII CCD diffractometer
Absorption correction: multi-scan (*SADABS*; Bruker, 2009 ▶) *T*_min_ = 0.267, *T*_max_ = 0.535
15470 measured reflections
4700 independent reflections
3640 reflections with *I* > 2σ(*I*)
*R*_int_ = 0.022

#### Refinement

*R*[*F* ^2^ > 2σ(*F* ^2^)] = 0.034
*wR*(*F* ^2^) = 0.086
*S* = 1.04
4700 reflections
181 parameters
H-atom parameters constrained
Δρ_max_ = 0.74 e Å^−3^
Δρ_min_ = −0.42 e Å^−3^

### 

Data collection: *APEX2* (Bruker, 2009 ▶); cell refinement: *SAINT* (Bruker, 2009 ▶); data reduction: *SAINT*; program(s) used to solve structure: *SHELXTL* (Sheldrick, 2008 ▶); program(s) used to refine structure: *SHELXTL*; molecular graphics: *SHELXTL*; software used to prepare material for publication: *SHELXTL* and *PLATON* (Spek, 2009 ▶).

## Supplementary Material

Crystal structure: contains datablock(s) global, I. DOI: 10.1107/S1600536811040311/hb6429sup1.cif

Structure factors: contains datablock(s) I. DOI: 10.1107/S1600536811040311/hb6429Isup2.hkl

Supplementary material file. DOI: 10.1107/S1600536811040311/hb6429Isup3.cml

Additional supplementary materials:  crystallographic information; 3D view; checkCIF report

## Figures and Tables

**Table 1:** Hydrogen-bond geometry (Å, °) *Cg*2 is the centroid of the C10–C15 ring.

*D*—H⋯*A*	*D*—H	H⋯*A*	*D*⋯*A*	*D*—H⋯*A*
O4—H1*O*4⋯O2^i^	0.83	1.97	2.7961 (19)	177
C12—H12*A*⋯Br1^ii^	0.95	2.90	3.7938 (18)	158
C14—H14*A*⋯O2^i^	0.95	2.52	3.198 (2)	129
C15—H15*A*⋯*Cg*2^i^	0.95	2.86	3.6181 (18)	137

Symmetry codes: (i) 
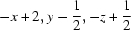
; (ii) 
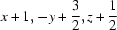
.

**Fig. 1.**
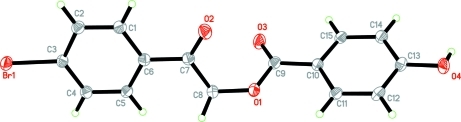
The molecular structure of the title compound, showing 50% probability displacement ellipsoids.

**Fig. 2.**
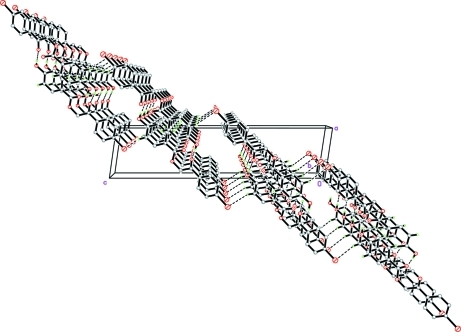
The crystal packing of the title compound, viewed along the showing the b axis. H atoms not involved in the intermolecular interactions (dashed lines) have been omitted for clarity.

## supplementary crystallographic information

### Comment

As part of our ongoing structural studies of phenacyl benzoates (Fun *et al.*, 2011), we now report the crystal structure of the title compound.

In the title compound (Fig. 1), the dihedral angle formed between the bromo-substituted (C1–C6) and hydroxy-substituted (C10–C15) benzene rings is 66.77 (8)°. Bond lengths and angles are within the normal ranges and are comparable to the related structure (Jin *et al.*, 2008).

In the crystal (Fig. 2), O4—H1O4···O2, C12—H12A···Br1 and C14—H14A···O2 hydrogen bonds (Table 1) link the molecules to form layers parallel to the (101) plane. The crystal packing is further consolidated by C—H···π interactions involving the centroid of the hydroxy-substituted benzene ring (*Cg*2; Table 1) and π–π interactions (Table 1) involving the centroids of the substituted benzene rings with the distance of *Cg*1···*Cg*2 being 3.5476 (7) Å. *Cg* 1 is the centroid of the bromo-substituted benzene ring.

### Experimental

A mixture of 4-hydroxybenzoic acid (1.0 g, 0.0072 mol), potassium carbonate (1.09 g, 0.0079 mol) and 2-bromo-1-(4-bromophenyl)ethanone (2.0 g, 0.0072 mol) in dimethylformamide (10 ml) was stirred at room temperature for 2 h. On cooling, colourless needle-shaped crystals of 2-(4-bromophenyl)-2-oxoethyl 4-hydroxybenzoate began to separate out. It was collected by filtration and recrystallized from ethanol to yield colourless blocks. Yield: 2.1 g, 86.7%. *M. p.*: 464–465 K (Lund & Langvad, 1932).

### Refinement

O– bound H atom was located from a difference Fourier map and was refined with a riding model with *U*_iso_(H) = 1.5 *U*_eq_(O) [O–H = 0.8286 Å]. The remaining H atoms were positioned geometrically and refined with a riding model with *U*_iso_(H) = 1.2 *U*_eq_(C) [C–H = 0.95 or 0.99 Å].

### Crystal data

**Table:**

C_15_H_11_BrO_4_	*F*(000) = 672
*M**_r_* = 335.15	*D*_x_ = 1.716 Mg m^−^^3^
Monoclinic, *P*2_1_/*c*	Mo *K*α radiation, λ = 0.71073 Å
Hall symbol: -P 2ybc	Cell parameters from 6519 reflections
*a* = 6.2917 (2) Å	θ = 2.7–32.6°
*b* = 7.7893 (2) Å	µ = 3.18 mm^−^^1^
*c* = 26.7497 (8) Å	*T* = 100 K
β = 98.234 (2)°	Block, colourless
*V* = 1297.43 (7) Å^3^	0.56 × 0.27 × 0.23 mm
*Z* = 4	

### Data collection

**Table:**

Bruker SMART APEXII CCD diffractometer	4700 independent reflections
Radiation source: fine-focus sealed tube	3640 reflections with *I* > 2σ(*I*)
graphite	*R*_int_ = 0.022
φ and ω scans	θ_max_ = 32.7°, θ_min_ = 1.5°
Absorption correction: multi-scan (*SADABS*; Bruker, 2009)	*h* = −9→9
*T*_min_ = 0.267, *T*_max_ = 0.535	*k* = −10→11
15470 measured reflections	*l* = −40→27

### Refinement

**Table:**

Refinement on *F*^2^	Primary atom site location: structure-invariant direct methods
Least-squares matrix: full	Secondary atom site location: difference Fourier map
*R*[*F*^2^ > 2σ(*F*^2^)] = 0.034	Hydrogen site location: inferred from neighbouring sites
*wR*(*F*^2^) = 0.086	H-atom parameters constrained
*S* = 1.04	*w* = 1/[σ^2^(*F*_o_^2^) + (0.0359*P*)^2^ + 1.236*P*] where *P* = (*F*_o_^2^ + 2*F*_c_^2^)/3
4700 reflections	(Δ/σ)_max_ = 0.001
181 parameters	Δρ_max_ = 0.74 e Å^−^^3^
0 restraints	Δρ_min_ = −0.42 e Å^−^^3^

### Special details

**Table:**

Experimental. The crystal was placed in the cold stream of an Oxford Cryosystems Cobra open-flow nitrogen cryostat (Cosier & Glazer, 1986) operating at 100.0 (1) K.
Geometry. All e.s.d.'s (except the e.s.d. in the dihedral angle between two l.s. planes) are estimated using the full covariance matrix. The cell e.s.d.'s are taken into account individually in the estimation of e.s.d.'s in distances, angles and torsion angles; correlations between e.s.d.'s in cell parameters are only used when they are defined by crystal symmetry. An approximate (isotropic) treatment of cell e.s.d.'s is used for estimating e.s.d.'s involving l.s. planes.
Refinement. Refinement of *F*^2^ against ALL reflections. The weighted *R*-factor *wR* and goodness of fit *S* are based on *F*^2^, conventional *R*-factors *R* are based on *F*, with *F* set to zero for negative *F*^2^. The threshold expression of *F*^2^ > σ(*F*^2^) is used only for calculating *R*-factors(gt) *etc*. and is not relevant to the choice of reflections for refinement. *R*-factors based on *F*^2^ are statistically about twice as large as those based on *F*, and *R*- factors based on ALL data will be even larger.

### Fractional atomic coordinates and isotropic or equivalent isotropic displacement parameters (Å^2^)

**Table:**

	*x*	*y*	*z*	*U*_iso_*/*U*_eq_	
Br1	−0.39495 (3)	0.75343 (3)	−0.055105 (7)	0.02365 (6)	
O1	0.4302 (2)	0.54838 (19)	0.21200 (5)	0.0227 (3)	
O2	0.4827 (2)	0.73017 (18)	0.12916 (5)	0.0238 (3)	
O3	0.6284 (2)	0.37103 (19)	0.17068 (5)	0.0247 (3)	
O4	1.2215 (2)	0.48897 (18)	0.38337 (5)	0.0226 (3)	
H1O4	1.3125	0.4144	0.3802	0.034*	
C1	0.1846 (3)	0.7654 (2)	0.04036 (7)	0.0188 (3)	
H1A	0.3228	0.8146	0.0404	0.023*	
C2	0.0288 (3)	0.7873 (2)	−0.00125 (7)	0.0200 (3)	
H2A	0.0596	0.8489	−0.0300	0.024*	
C3	−0.1742 (3)	0.7171 (2)	−0.00004 (7)	0.0179 (3)	
C4	−0.2212 (3)	0.6222 (2)	0.04075 (7)	0.0196 (3)	
H4A	−0.3600	0.5740	0.0406	0.024*	
C5	−0.0626 (3)	0.5984 (2)	0.08189 (7)	0.0193 (3)	
H5A	−0.0923	0.5322	0.1099	0.023*	
C6	0.1407 (3)	0.6715 (2)	0.08233 (7)	0.0173 (3)	
C7	0.3110 (3)	0.6554 (2)	0.12660 (7)	0.0180 (3)	
C8	0.2639 (3)	0.5418 (3)	0.16963 (7)	0.0221 (4)	
H8A	0.1268	0.5784	0.1804	0.027*	
H8B	0.2464	0.4218	0.1575	0.027*	
C9	0.6069 (3)	0.4528 (2)	0.20814 (7)	0.0192 (3)	
C10	0.7646 (3)	0.4610 (2)	0.25472 (6)	0.0172 (3)	
C11	0.7237 (3)	0.5494 (2)	0.29804 (7)	0.0192 (3)	
H11A	0.5895	0.6051	0.2984	0.023*	
C12	0.8786 (3)	0.5556 (2)	0.34023 (7)	0.0205 (3)	
H12A	0.8503	0.6154	0.3695	0.025*	
C13	1.0759 (3)	0.4745 (2)	0.34009 (7)	0.0194 (3)	
C14	1.1169 (3)	0.3844 (2)	0.29743 (7)	0.0203 (3)	
H14A	1.2508	0.3283	0.2972	0.024*	
C15	0.9606 (3)	0.3775 (2)	0.25526 (7)	0.0188 (3)	
H15A	0.9877	0.3148	0.2264	0.023*	

### Atomic displacement parameters (Å^2^)

**Table:**

	*U*^11^	*U*^22^	*U*^33^	*U*^12^	*U*^13^	*U*^23^
Br1	0.02023 (9)	0.03119 (11)	0.01835 (9)	−0.00343 (7)	−0.00127 (6)	0.00545 (8)
O1	0.0199 (6)	0.0322 (8)	0.0155 (6)	0.0060 (5)	0.0008 (5)	0.0007 (5)
O2	0.0200 (6)	0.0264 (7)	0.0237 (6)	−0.0039 (5)	−0.0012 (5)	−0.0001 (5)
O3	0.0268 (6)	0.0281 (7)	0.0179 (6)	0.0050 (6)	−0.0012 (5)	−0.0049 (5)
O4	0.0223 (6)	0.0244 (7)	0.0192 (6)	0.0043 (5)	−0.0032 (5)	0.0036 (5)
C1	0.0184 (7)	0.0190 (8)	0.0190 (7)	−0.0024 (6)	0.0024 (6)	−0.0014 (7)
C2	0.0210 (8)	0.0200 (9)	0.0190 (8)	−0.0014 (6)	0.0027 (6)	0.0020 (6)
C3	0.0179 (7)	0.0201 (9)	0.0151 (7)	−0.0003 (6)	0.0002 (6)	−0.0009 (6)
C4	0.0179 (7)	0.0221 (9)	0.0184 (8)	−0.0017 (6)	0.0014 (6)	0.0000 (7)
C5	0.0195 (7)	0.0218 (9)	0.0165 (7)	−0.0013 (6)	0.0024 (6)	0.0023 (6)
C6	0.0184 (7)	0.0168 (8)	0.0167 (8)	0.0007 (6)	0.0022 (6)	−0.0012 (6)
C7	0.0181 (7)	0.0184 (8)	0.0177 (8)	0.0015 (6)	0.0026 (6)	−0.0022 (6)
C8	0.0178 (7)	0.0297 (10)	0.0183 (8)	0.0015 (7)	0.0004 (6)	0.0025 (7)
C9	0.0211 (8)	0.0194 (9)	0.0170 (7)	0.0006 (6)	0.0031 (6)	0.0022 (6)
C10	0.0185 (7)	0.0176 (8)	0.0153 (7)	0.0011 (6)	0.0018 (6)	0.0001 (6)
C11	0.0220 (8)	0.0202 (9)	0.0160 (8)	0.0034 (6)	0.0044 (6)	0.0019 (6)
C12	0.0285 (9)	0.0196 (9)	0.0138 (7)	0.0033 (7)	0.0041 (6)	0.0002 (6)
C13	0.0242 (8)	0.0177 (8)	0.0154 (7)	0.0004 (6)	0.0002 (6)	0.0019 (6)
C14	0.0209 (8)	0.0212 (9)	0.0185 (8)	0.0033 (6)	0.0023 (6)	−0.0004 (7)
C15	0.0217 (8)	0.0201 (8)	0.0149 (7)	0.0017 (6)	0.0037 (6)	−0.0004 (6)

### Geometric parameters (Å, °)

**Table:**

Br1—C3	1.8954 (17)	C5—H5A	0.9500
O1—C9	1.354 (2)	C6—C7	1.484 (2)
O1—C8	1.429 (2)	C7—C8	1.514 (3)
O2—C7	1.221 (2)	C8—H8A	0.9900
O3—C9	1.211 (2)	C8—H8B	0.9900
O4—C13	1.374 (2)	C9—C10	1.479 (2)
O4—H1O4	0.8286	C10—C15	1.393 (2)
C1—C2	1.384 (3)	C10—C11	1.403 (2)
C1—C6	1.400 (2)	C11—C12	1.382 (3)
C1—H1A	0.9500	C11—H11A	0.9500
C2—C3	1.395 (2)	C12—C13	1.394 (3)
C2—H2A	0.9500	C12—H12A	0.9500
C3—C4	1.385 (3)	C13—C14	1.395 (3)
C4—C5	1.388 (2)	C14—C15	1.387 (2)
C4—H4A	0.9500	C14—H14A	0.9500
C5—C6	1.399 (2)	C15—H15A	0.9500
			
C9—O1—C8	115.84 (15)	C7—C8—H8A	109.1
C13—O4—H1O4	104.1	O1—C8—H8B	109.1
C2—C1—C6	120.74 (16)	C7—C8—H8B	109.1
C2—C1—H1A	119.6	H8A—C8—H8B	107.9
C6—C1—H1A	119.6	O3—C9—O1	122.84 (17)
C1—C2—C3	118.57 (17)	O3—C9—C10	125.39 (17)
C1—C2—H2A	120.7	O1—C9—C10	111.77 (15)
C3—C2—H2A	120.7	C15—C10—C11	119.19 (16)
C4—C3—C2	121.84 (16)	C15—C10—C9	118.33 (16)
C4—C3—Br1	118.48 (13)	C11—C10—C9	122.48 (16)
C2—C3—Br1	119.67 (14)	C12—C11—C10	120.00 (17)
C3—C4—C5	119.05 (16)	C12—C11—H11A	120.0
C3—C4—H4A	120.5	C10—C11—H11A	120.0
C5—C4—H4A	120.5	C11—C12—C13	120.42 (17)
C4—C5—C6	120.35 (17)	C11—C12—H12A	119.8
C4—C5—H5A	119.8	C13—C12—H12A	119.8
C6—C5—H5A	119.8	O4—C13—C12	116.55 (16)
C5—C6—C1	119.40 (16)	O4—C13—C14	123.48 (16)
C5—C6—C7	121.80 (16)	C12—C13—C14	119.96 (16)
C1—C6—C7	118.78 (16)	C15—C14—C13	119.49 (17)
O2—C7—C6	122.43 (17)	C15—C14—H14A	120.3
O2—C7—C8	120.28 (16)	C13—C14—H14A	120.3
C6—C7—C8	117.30 (15)	C14—C15—C10	120.91 (16)
O1—C8—C7	112.40 (15)	C14—C15—H15A	119.5
O1—C8—H8A	109.1	C10—C15—H15A	119.5
			
C6—C1—C2—C3	1.4 (3)	C8—O1—C9—O3	2.6 (3)
C1—C2—C3—C4	−2.1 (3)	C8—O1—C9—C10	−177.10 (15)
C1—C2—C3—Br1	177.19 (14)	O3—C9—C10—C15	4.2 (3)
C2—C3—C4—C5	0.9 (3)	O1—C9—C10—C15	−176.10 (16)
Br1—C3—C4—C5	−178.33 (14)	O3—C9—C10—C11	−175.77 (19)
C3—C4—C5—C6	0.9 (3)	O1—C9—C10—C11	3.9 (3)
C4—C5—C6—C1	−1.5 (3)	C15—C10—C11—C12	1.2 (3)
C4—C5—C6—C7	177.18 (17)	C9—C10—C11—C12	−178.79 (18)
C2—C1—C6—C5	0.4 (3)	C10—C11—C12—C13	0.1 (3)
C2—C1—C6—C7	−178.38 (17)	C11—C12—C13—O4	179.19 (17)
C5—C6—C7—O2	−173.81 (18)	C11—C12—C13—C14	−0.9 (3)
C1—C6—C7—O2	4.9 (3)	O4—C13—C14—C15	−179.65 (17)
C5—C6—C7—C8	5.8 (3)	C12—C13—C14—C15	0.5 (3)
C1—C6—C7—C8	−175.45 (16)	C13—C14—C15—C10	0.8 (3)
C9—O1—C8—C7	−79.3 (2)	C11—C10—C15—C14	−1.7 (3)
O2—C7—C8—O1	5.1 (3)	C9—C10—C15—C14	178.32 (17)
C6—C7—C8—O1	−174.58 (15)		

### Hydrogen-bond geometry (Å, °)

**Table:**

Cg2 is the centroid of the C10–C15 ring.

**Table:**

*D*—H···*A*	*D*—H	H···*A*	*D*···*A*	*D*—H···*A*
O4—H1O4···O2^i^	0.83	1.97	2.7961 (19)	177
C12—H12A···Br1^ii^	0.95	2.90	3.7938 (18)	158
C14—H14A···O2^i^	0.95	2.52	3.198 (2)	129
C15—H15A···Cg2^i^	0.95	2.86	3.6181 (18)	137

Symmetry codes: (i) −*x*+2, *y*−1/2, −*z*+1/2; (ii) *x*+1, −*y*+3/2, *z*+1/2.
